# Association of tubular injury with lipid metabolism: A Mendelian randomization study

**DOI:** 10.1097/MD.0000000000046279

**Published:** 2025-12-19

**Authors:** Keqin Zhao, Linlin Qian, Xiaobei Ma

**Affiliations:** a Institute of Basic Theory for Chinese Medicine, China Academy of Chinese Medical Sciences, Beijing, China; b Graduate School, China Academy of Chinese Medical Sciences, Beijing, China.

**Keywords:** causal association, KIM-1, lipid profiles, Mendelian randomization, renal tubular injury

## Abstract

Patients with chronic kidney disease frequently exhibit abnormalities in their lipid metabolism. Confounding factors in observational studies often obscure the causal relationship between these 2 diseases. This study investigated the causal relationships between genetically predicted levels of 6 key lipid parameters (total cholesterol (TC), triglycerides (TG), HDL-C, low-density lipoprotein cholesterol (LDL-C), apolipoprotein A1 (ApoA1), and apolipoprotein B (ApoB)) and circulating kidney injury molecule 1 (KIM-1) levels, using a comprehensive bidirectional Mendelian randomization (MR) analysis. Using genome-wide association study data, the primary analysis used the inverse-variance weighted (IVW) method, supported by MR-Egger regression and a weighted median estimator. Sensitivity analyses including heterogeneity, pleiotropy tests, leave-one-out, and reverse causality analyses were conducted. The IVW model revealed the following: TG (odds ratio (OR): 1.1843, 95% confidence interval (CI): 1.1178–1.2547, *P* = 9.5894e−09), TC (OR: 1.1096, 95% CI: 1.0178–1.2095, *P* = .0182), and ApoA1 (OR: 1.1820, 95% CI: 1.0741–1.3007, *P* = .0007) were found to have significant causal relationships with KIM-1, a biomarker of kidney tubular injury, and may be risk factors for renal tubular injury; No significant causal associations were observed between high-density lipoprotein cholesterol (HDL-C), (*P* = .2929), LDL-C (*P* = .2178), ApoB (*P* = .1836), and KIM-1; Horizontal pleiotropy was detected for ApoA1 (*P* = .0208). However, sensitivity analyses confirmed the robustness of the results after the removal of outliers; significant heterogeneity was observed across all lipid parameters (Cochran *Q P* < .05), which necessitated the use of random-effects IVW models; and reverse causality analyses (MR-Egger intercept *P* > .05, Steiger filtering) confirmed no evidence of reverse causation between lipid profiles and KIM-1. TG, HDL-C, and ApoA1 levels may be risk factors for renal tubular injury. However, no significant causal relationships were observed between HDL-C, LDL-C, and ApoB levels and renal tubular injury. To further explore the underlying mechanisms of the associations between TG, HDL-C, ApoA1, and KIM-1 and to inform lipid management strategies in tubulopathy-related conditions.

## 1. Introduction

Chronic kidney disease (CKD) represents a significant and escalating global health burden, intricately linked with the rising prevalence of metabolic disorders, such as type 2 diabetes and dyslipidemia.^[1,2]^ CKD progression often follows a silent trajectory in its early stages, underscoring the critical need for sensitive biomarkers capable of detecting incipient kidney damage before irreversible functional decline occurs.^[3,4]^ Among these, kidney injury molecule-1 (KIM-1), a transmembrane protein that is markedly upregulated in injured proximal tubular epithelial cells, has emerged as a highly specific and sensitive noninvasive biomarker for detecting acute and chronic kidney tubular injury, a key pathological event in the initiation and progression of various nephropathies, including diabetic nephropathy.^[5,6]^

Dyslipidemia is characterized by aberrant levels of circulating lipids and apolipoproteins, including total cholesterol (TC), triglycerides (TG), high-density lipoprotein cholesterol (HDL-C), low-density lipoprotein cholesterol (LDL-C), apolipoprotein A1 (ApoA1), and apolipoprotein B (ApoB). It is a well-established risk factor for cardiovascular disease and is frequently observed in patients with CKD.^[7]^ Observational studies have consistently reported associations between specific lipid profiles and markers of kidney dysfunction or CKD progression.^[8–10]^ However, establishing definitive causal relationships based on these observational findings remains challenging. Such associations are susceptible to confounding by shared risk factors (e.g., obesity, hypertension, and glycemic control) and the potential for reverse causality, in which impaired kidney function itself might alter lipid metabolism.^[11,12]^ Consequently, the precise causal role of individual lipid fractions and their associated apolipoproteins in initiating or promoting kidney tubular injury, as reflected by KIM-1 levels, remains largely unknown.

Mendelian randomization (MR) offers a robust analytical approach to overcome the inherent limitations of observational studies and dissect the potential causal nexus, MR offers a robust analytical approach.^[13–15]^ By employing genetic variants strongly associated with exposure (lipid levels) as instrumental variables (IVs), MR minimizes confounding and is less prone to reverse causation bias, thereby strengthening causal inference in complex etiological pathways.^[16,17]^ While previous MR studies have explored links between lipid traits and broader kidney function metrics or CKD diagnosis, a critical gap persists.^[18,19]^ Specifically, the causal effects of a comprehensive panel of key lipids and their major apolipoproteins (TC, TG, HDL-C, LDL-C, ApoA1, and ApoB) on a sensitive early marker of tubular injury, KIM-1, have not been systematically investigated using this powerful causal inference framework. Furthermore, assessing the bidirectionality of this relationship – whether kidney tubular injury might, in turn, influence lipid profiles – requires a dedicated bidirectional MR design, an approach yet to be applied to this specific set of interactions. Therefore, leveraging large-scale genome-wide association study (GWAS) summary statistics within a rigorous bidirectional MR framework presents an unparalleled opportunity to clarify these complex causal relationships.

In this study, we conducted, for the first time, a comprehensive bidirectional MR analysis to investigate the causal relationships between genetically predicted levels of 6 key lipid parameters (TC, TG, HDL-C, LDL-C, ApoA1, and ApoB) and circulating KIM-1 levels. Utilizing summary statistics from large-scale GWAS datasets, we employed robust MR methods, including inverse-variance weighted (IVW), MR-Egger, and weighted median estimators (WMEs), complemented by extensive sensitivity analyses, to ensure the validity of our findings. Our results provide compelling genetic evidence suggesting that elevated TG levels causally increase the risk of kidney tubular injury (indicated by higher KIM-1 levels), whereas higher levels of HDL-C and ApoA1 exert a protective causal effect. Conversely, no significant causal links were identified between TC, LDL-C, or ApoB and KIM-1 levels, and importantly, we found no evidence supporting reverse causation. This study elucidates the distinct causal roles of specific lipid components in early kidney injury pathogenesis, offering novel insights that could inform targeted lipid management strategies for preserving kidney health, particularly in populations at high risk of metabolic syndrome and related complications. The subsequent sections detail our methodology, present the main findings and sensitivity analyses, and discuss the implications of these results within the broader context of diabetic nephropathy and metabolic disease management.

## 2. Materials and methods

### 2.1. Data resources

In this study, the exposure factors were set as the levels of 6 lipid profile parameters (TC, TG, HDL-C, LDL-C, ApoA1, and ApoB) and the outcome variable was KIM-1. These parameters were used to explore the different aspects of kidney function impairment, enabling a comprehensive evaluation.^[20]^ The GWAS data for TC (met-d-Total_C), TG (ieu-b-111), LDL-C (ieu-b-110), HDL-C (ieu-b-4844), ApoA1 (ieu-b-107), ApoB (ieu-b-108), and KIM-1 (ebi-a-GCST90012041) were obtained from the IEU OpenGWAS project (mrcieu.ac.uk). The sample sizes for the lipid profile datasets were as follows: TC, 115,078; TG, 441,016; LDL-C, 440,546; HDL-C, 77,409; ApoA1, 393,193; and ApoB, 439,214. The single nucleotide polymorphism (SNP) counts were 12,321,875 for TC, 12,321,875 for TG, 12,321,875 for LDL-C, 7892,377 for HDL-C, 12,321,875 for ApoA1, and 12,321,875 for ApoB. For KIM-1, the sample size was 21,758, with an SNP count of 13,103,458.

Extensive genomic research and the significant accumulation of SNP data in European populations, along with the international validation and application of these findings, have led to many GWAS and MR studies relying on these data. Thus, this study primarily used data from European populations, with all studies approved by the relevant ethics review boards. As this study relied on publicly available data, approval from an institutional ethics review board was not necessary.

### 2.2. IVs selection

SNPs associated with 6 lipid profile parameters were identified as IVs.^[21]^ The selection process entailed several key steps: mitigating bias from linkage disequilibrium. To avoid potential bias stemming from strong linkage disequilibrium among SNPs, we selected independent SNPs that demonstrated significant genome-wide associations with the 6 lipid parameters to serve as IVs. The criteria for selection were as follows: *P*-value < 5 × 10^−8^, *r*^2^ = 0.001, and a spatial separation of 10,000 kilobases (kb). This approach ensured the independence of each SNP and reduced the impact of pleiotropy on findings.^[22,23]^ By filtering SNPs from GWAS Utilizing the GWAS data for KIM-1, we selected SNPs that exhibited associations with the exposure of interest. Missing SNPs were substituted with highly correlated SNPs and those without replacements were removed.^[24]^ Merging datasets: Datasets pertaining to the 6 lipid parameters were combined with those related to KIM-1. SNPs that were directly linked to KIM-1 (*P* < 5 × 10^−8^) were eliminated.^[25]^ The final dataset included the requisite IVs for this analysis.

### 2.3. Principles of MR study

**Figure 1.**
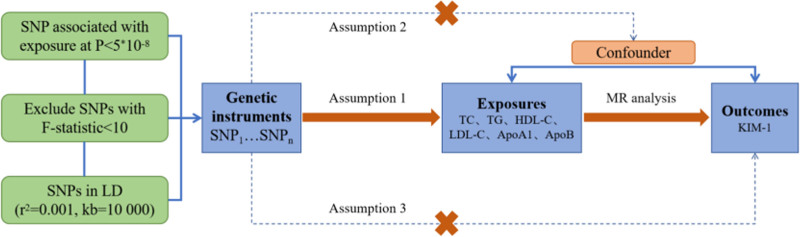
Three major assumptions of MR studies. MR = Mendelian randomization.

The study design rigorously adheres to the 3 fundamental assumptions of MR research (Fig. 1): Relevance Assumption: The IVs are strongly associated with the exposure factors; Independence Assumption: The IVs were independent of other confounding factors; and Exclusivity Assumption: The Genetic variants influence the outcome exclusively through exposure factors. To test the relevance assumption, the explanatory power of the IVs for the exposure factors was evaluated using an *F*-statistic. An *F*-statistic >10 indicates that the likelihood of weak instrument bias, which could violate the relevance assumption, is minimal. Sensitivity analyses were conducted to assess the heterogeneity and pleiotropy. This study was conducted and documented in alignment with the Strengthening the Reporting of Observational Studies in Epidemiology using Mendelian randomization guidelines.^[26]^

### 2.4. MR analysis

Three regression models – MR-Egger regression, WME, and the IVW method – were employed in this study. Using SNPs as IVs, these models assessed the causal relationships between the 6 lipid profile parameters, DN, and urinary albumin-to-creatinine ratio. These MR methods test the robustness and credibility of the causal relationships under different assumptions and are the most commonly used methods in MR studies, providing consistent estimates of causal effects with high statistical power and the most accurate causal effect estimates. However, it is highly sensitive to pleiotropy, and the presence of pleiotropy may lead to biased estimates. MR-Egger, while less powerful than IVW in terms of testing efficacy, can detect and correct pleiotropic bias, making it useful for sensitivity analysis when pleiotropy is suspected. The WME provides consistent causal effect estimates by assigning more weight to valid IVs, but it has lower power for detecting causal effects. Therefore, in this study, IVW was chosen as the primary method for causal relationship assessment, with MR-Egger and WME serving as the supplementary methods. Additionally, attention should be paid to the 95% confidence interval (CI) of the odds ratio (OR). When the 95% CI of the OR did not include 1, it typically indicated statistical significance. If the CI includes 1, it suggests a lack of a significant association. If the results from different instruments show significant discrepancies, a heterogeneity analysis may be required.

### 2.5. Sensitivity analysis

Several sensitivity analyses were conducted to ensure the robustness of the MR results. Horizontal pleiotropy detection: MR-Egger intercept analysis was used to detect horizontal pleiotropy. If the intercept term from the MR-Egger intercept analysis is statistically significant compared to zero, it indicates the presence of horizontal pleiotropy in the study^[27]^; Heterogeneity assessment: Cochran *Q* test was used to evaluate the heterogeneity among SNPs. A statistically significant Cochran *Q* statistic indicated notable heterogeneity among SNPs, warranting particular attention to the results from the random-effects IVW method^[28]^; Leave-one-out sensitivity test: This test assesses the robustness of the MR results by sequentially excluding each SNP and recalculating them. If the recalculated MR results were not substantially different from the complete MR results, the MR results were considered robust.

### 2.6. Reverse causality detection

To detect potential reverse causality, SNPs were selected for bidirectional MR analysis via GWAS data. The causal effects were estimated via the MR-IVW, MR-Egger, weighted median, simple mode, and weighted mode methods. Steiger filtering was conducted to ensure the directionality of the association between the lipid profiles and DN. The results were considered statistically significant at *P* < .05.

All data analyses in this study were conducted using the TwoSampleMR package in the R software (version 4.3.2).

## 3. Results

### 3.1. The 6 lipid profiles and KIM-1

According to the IV selection criteria of this study, the following SNPs were identified for the 6 lipid profile parameters when the outcome variable was KIM-1: The MR-Egger regression intercepts were as follows: *b*_TC_ = 0.0047 (*P* = .2630), *b*_TG_ = 0.0009 (*P* = .5310), *b*_LDL-C_ = 0.0005 (*P* = .8054), *b*_HDL-C_ = −0.0050 (*P* = .2929), *b*_ApoA1_ = −0.0033 (*P* = .0208), and *b*_ApoB_ = 0.0032 (*P* = .1000). No evidence of horizontal pleiotropy was observed for SNPs associated with KIM-1 outcome, with the exception of ApoA1. This finding suggests that the MR method is effective for causal inference in this context, with the exception of ApoA1. Specifically, for horizontal pleiotropy of ApoA1, 2 outliers were identified using the Mendelian randomization pleiotropy residual sum and outlier method. However, the removal of these outliers did not result in a significant difference in the results, indicating that the overall findings remained robust (Table 1).

**Table 1:** Intercept test of the MR-Egger regression model for 6 lipid profile parameters.

Exposure	ID	Outcome	ID	SNPs	Intercept term *b*	*P*-value
TC	met-d-Total_C	Kidney injury molecule-1 (KIM-1)	ebi-a-GCST90012041	59	0.0047	.2630
TG	ieu-b-111			313	0.0009	.5310
LDL-C	ieu-b-110			164	0.0005	.8054
HDL-C	ieu-b-4844			78	−0.0050	.2929
ApoA1	ieu-b-107			280	−0.0033	.0208
ApoB	ieu-b-108			187	.0032	.1000

ApoA1 = apolipoprotein A1, ApoB = apolipoprotein B, HDL-C = high-density lipoprotein cholesterol, LDL-C = low-density lipoprotein cholesterol, MR = Mendelian randomization, SNPs = single nucleotide polymorphisms, TC = total cholesterol, TG = triglycerides.

### 3.2. MR analysis

**Figure 2.**
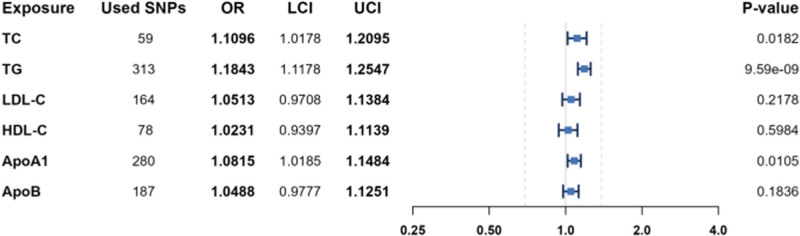
Causal effects of 6 lipid profile parameters on KIM-1. KIM-1 = kidney injury molecule 1.

The regression results of the 3 methods are shown in Table 2 and Figure 2 Causal effects of 6 lipid profile parameters on KIM-1. Model IVW revealed that: Model IVW revealed that TC (OR: 1.1096, 95% CI: 1.0178–1.2095, *P* = .0182), TG (OR: 1.1843, 95% CI: 1.1178–1.2547, *P* = 9.5894e−09), and ApoA1 (OR: 1.0815, 95% CI: 1.0185–1.1484, *P* = .0105) were causally associated with KIM-1; and LDL-C, HDL-C, and ApoB showed no significant causal association with KIM-1 (all *P* > .05). The results indicate that elevated TG, ApoA1, and HDL-C may have causal relationships with KIM-1, whereas TC, LDL-C, and ApoB do not exhibit significant causal effects.

**Table 2:** Regression results of 3 MR methods for 6 lipid profile parameters.

Outcome	Exposure	Method	β	SE	OR (95% CI)	*P*-value
KIM-1	TC	MR-Egger	0.0385	0.0727	1.0392 (0.9013–1.1983)	.5986
		WME	0.0081	0.0483	1.0081 (0.9170–1.1083)	.8668
		IVW	0.1040	0.0440	1.1096 (1.0178–1.2095)	.0182
	TG	MR-Egger	0.1482	0.0445	1.1598 (1.0629–1.2655)	.0010
		WME	0.0981	0.0480	1.1031 (1.0041–1.2118)	.0408
		IVW	0.1691	0.0295	1.1843 (1.1178–1.2547)	9.5894e−09
	LDL-C	MR-Egger	0.0386	0.0616	1.0393 (0.9210–1.1729)	.5322
		WME	−0.0337	0.0474	0.9668 (0.8810–1.0610)	.4773
		IVW	0.0500	0.0406	1.0513 (0.9708–1.1384)	.2178
	HDL-C	MR-Egger	0.0913	0.0778	1.0956 (0.9407–1.2761)	.2446
		WME	0.0361	0.0455	1.0367 (0.9482–1.1335)	.4282
		IVW	0.0228	0.0434	1.0231 (0.9397–1.1139)	.5984
	ApoA1	MR-Egger	0.1672	0.0488	1.1820 (1.0741–1.3007)	.0007
		WME	0.1474	0.0446	1.1588 (1.0618–1.2648)	.0010
		IVW	0.0783	0.0306	1.0815 (1.0185–1.1484)	.0105
	ApoB	MR-Egger	−0.0091	0.0495	0.9909 (0.8993–1.0919)	.8539
		WME	−0.0264	0.0419	0.9740 (0.8971–1.0573)	.5289
		IVW	0.0477	0.0358	1.0488 (0.9777–1.1251)	.1836

ApoA1 = apolipoprotein A1, ApoB = apolipoprotein B, CI = confidence interval, HDL-C = high-density lipoprotein cholesterol, IVW = inverse-variance weighted, KIM-1 = kidney injury molecule 1, LDL-C = low-density lipoprotein cholesterol, MR = Mendelian randomization, OR = odds ratio, TC = total cholesterol, TG = triglycerides, WME = weighted median estimator.

Table 1 Regression results of 3 MR methods for 6 lipid profile parameters.

### 3.3. Heterogeneity test

**Figure 3.**
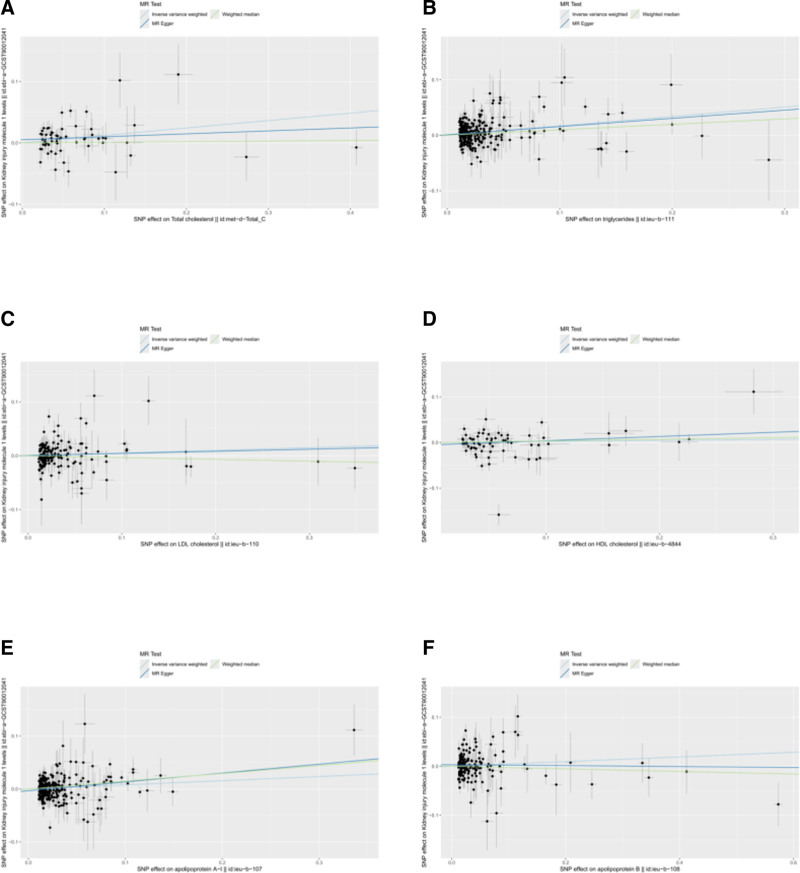
Scatter plots of MR analysis for 6 lipid profile parameters. Panels A, B, C, D, E, and F correspond to TC, TG, LDL-C, HDL-C, ApoA1, and ApoB, respectively. ApoA1 = apolipoprotein A1, ApoB = apolipoprotein B, HDL-C = high-density lipoprotein cholesterol, LDL-C = low-density lipoprotein cholesterol, MR = Mendelian randomization, TC = total cholesterol, TG = triglycerides.

**Figure 4.**
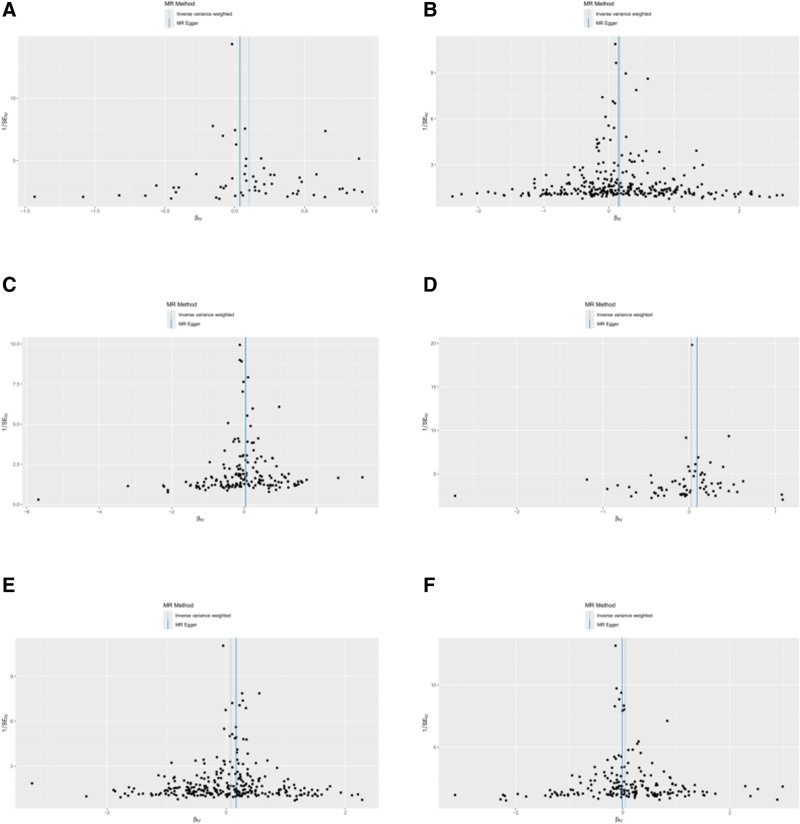
Funnel plots of MR analysis for 6 lipid profile parameters. Panels A, B, C, D, E, and F correspond to TC, TG, LDL-C, HDL-C, ApoA1, and ApoB, respectively. ApoA1 = apolipoprotein A1, ApoB = apolipoprotein B, HDL-C = high-density lipoprotein cholesterol, LDL-C = low-density lipoprotein cholesterol, MR = Mendelian randomization, TC = total cholesterol, TG = triglycerides.

Scatter and funnel plots are shown in Figures 3 and 4. The funnel plot demonstrated essential symmetry among all included SNPs, indicating that the causal effect inferred using SNPs as IVs was minimally influenced by potential bias. However, the results of the Cochran *Q* test revealed significant heterogeneity among the included IVs for all lipid parameters (*P* < .05) (Table 3). Therefore, the findings from the random-effects IVW model should be emphasized to account for this heterogeneity.

**Table 3:** Cochran *Q* test results for 6 lipid profile parameters.

Outcome	Exposure	Cochran *Q* result	*P*-value
KIM-1	TC	130.163	4.232459e−07
	TG	399.185	3.545739e−05
	LDL-C	303.116	1.698163e−10
	HDL-C	188.169	1.912742e−13
	ApoA1	391.906	9.275329e−06
	ApoB	360.1373	3.224302e−13

ApoA1 = apolipoprotein A1, ApoB = apolipoprotein B, HDL-C = high-density lipoprotein cholesterol, KIM-1 = kidney injury molecule 1, LDL-C = low-density lipoprotein cholesterol, MR = Mendelian randomization, TC = total cholesterol, TG = triglycerides.

### 3.4. Sensitivity analysis

By analyzing the SNPs of TC, TG, HDL, LDL, ApoA1, and ApoB, it was found that the distribution of these indicators on the MR effect size was highly consistent. The effect size and CI of each SNP were roughly concentrated around the overall effect size, with no significant trend of deviation from the overall effect value. This consistency indicates that these IVs are robust in evaluating the relationship between lipid-related exposures and KIM-1 levels, further supporting the neutral or weak effect of these exposures on KIM-1 levels. These results suggest that the IVs used in this study are representative and not significantly influenced by a single genetic variant.

Sensitivity analysis was conducted using the leave-one-out method for lipid-related exposures, including TC, TG, HDL, LDL, ApoA1, and ApoB, in relation to KIM-1 levels. The results showed that the overall findings for these exposures were not driven by any single SNP. The consistency across all analyses indicates that the Mendelian randomization results in this study are robust and not influenced by a single genetic variant.

### 3.5. Reverse causality detection

Reverse MR-Egger analysis suggested that all identified plasma proteins exhibited reliable directionality (*P* > .05). Additionally, Reverse MR analysis did not detect any possible reverse confounding factors, further confirming robust directionality, as detailed in Tables 4 and 5.

**Table 4:** MR-Egger intercept test for KIM-1.

Exposure	ID	Outcome	ID	SNPs	Intercept term *b*	*P*-value
KIM-1	ebi-a-GCST90012041	TC	met-d-Total_C	12	0.0160	.1897
		TG	ieu-b-111	13	0.0283	.0591
		LDL-C	ieu-b-110	13	0.0201	.0365
		HDL-C	ieu-b-4844	13	−0.0071	.2135
		ApoA1	ieu-b-107	13	0.0158	.3846
		ApoB	ieu-b-108	13	0.0225	.0446

ApoA1 = apolipoprotein A1, ApoB = apolipoprotein B, HDL-C = high-density lipoprotein cholesterol, KIM-1 = kidney injury molecule 1, LDL-C = low-density lipoprotein cholesterol, MR = Mendelian randomization, SNPs = single nucleotide polymorphisms, TC = total cholesterol, TG = triglycerides.

**Table 5:** Regression results from 3 MR methods for KIM-1.

Outcome	Exposure	Method	β	SE	OR (95% CI)	*P*-value
TC	KIM-1	MR-Egger	−0.0479	0.0530	0.9533 (0.8591–1.0577)	.3881
		WME	−0.0139	0.0101	0.9862 (0.9669–1.0059)	.1689
		IVW	0.0038	0.0399	1.0039 (0.9282–1.0856)	.9233
TG		MR-Egger	−0.0735	0.0675	0.9291 (0.8139–1.0605)	.2993
		WME	−0.0127	0.0049	0.9873 (0.9779–0.9968)	.0091
		IVW	0.0232	0.0561	1.0235 (0.9170–1.1423)	.6788
LDL-C		MR-Egger	−0.0576	0.0425	0.9440 (0.8686–1.0260)	.2021
		WME	−0.0157	0.0052	0.9844 (0.9743–0.9946)	.0028
		IVW	0.0112	0.0366	1.0113 (0.9412–1.0866)	.7595
HDL-C		MR-Egger	0.0121	0.0239	1.0123 (0.9658–1.0610)	.6234
		WME	−0.0067	0.0112	0.9933 (0.9716–1.0155)	.5505
		IVW	−0.0103	0.0178	0.9897 (0.9559–1.0248)	.5619
ApoA1		MR-Egger	−0.0354	0.0877	0.9652 (0.8127–1.1462)	.6939
		WME	−0.0021	0.0044	0.9978 (0.9893–1.0064)	.6169
		IVW	0.0187	0.0637	1.0189 (0.8992–1.1544)	.7693
ApoB		MR-Egger	−0.0639	0.0499	0.9381 (0.8507–1.0345)	.2270
		WME	−0.0160	0.0047	0.9842 (0.9752–0.9933)	.0007
		IVW	0.0132	0.0424	1.0133 (0.9325–1.1010)	.7560

ApoA1 = apolipoprotein A1, ApoB = apolipoprotein B, CI = confidence interval, HDL-C = high-density lipoprotein cholesterol, IVW = inverse-variance weighted, KIM-1 = kidney injury molecule 1, LDL-C = low-density lipoprotein cholesterol, MR = Mendelian randomization, OR = odds ratio, TC = total cholesterol, TG = triglycerides, WME = weighted median estimator.

## 4. Discussion

This study, for the first time, leverages Mendelian randomization to provide genetic evidence for a causal relationship between elevated levels of KIM-1, an early biomarker of tubular injury, and high levels of TG, TC, and ApoA1. However, in this study, the causal associations between LDL-C, HDL-C, ApoB, and KIM-1 levels did not reach statistical significance. Notably, in this analysis, LDL-C and HDL-C did not show direct causal associations with KIM-1, indicating that the mechanisms by which lipids affect early kidney injury may be more complex than previously thought. Metabolic pathways related to TC, TG, and ApoA1 may play a more direct role in the early stages of tubular injury. This finding provides a new direction for the future exploration of the pathogenesis of kidney disease and the search for new therapeutic targets.

KIM-1 is a member of the type-I transmembrane glycoprotein family.^[29]^ Under physiological conditions, the expression of KIM-1 in mature kidney tissue is suppressed, and its presence is difficult to detect using conventional methods.^[30]^ Notably, upon pathological insults, such as ischemia-reperfusion injury, exposure to toxic substances, inflammatory responses, or metabolic imbalances, KIM-1 is significantly upregulated in a spatiotemporally specific manner in the apical membrane of renal tubular epithelial cells, particularly in proximal tubule epithelial cells. Studies have shown that KIM-1 mRNA levels increase significantly within 30 minutes of ischemia-reperfusion (3-fold) and reach a peak at 24 hours (9-fold), with KIM-1 primarily localized in the apical membrane of proximal tubule epithelial cells (PTECs), highly overlapping with damaged areas.^[31,32]^ Numerous clinical studies have confirmed that KIM-1, as a novel biomarker, has dual advantages: first, its tissue-specific expression pattern makes it a highly sensitive indicator of renal tubular injury^[33]^; second, urinary KIM-1 levels dynamically change in the preclinical phase, with abnormal increases occurring earlier than significant alterations in traditional renal function parameters such as serum creatinine (Scr) and blood urea nitrogen, demonstrating an important early warning value.^[34]^

TC primarily reflects the metabolic state of cholesterol-enriched lipoproteins, particularly LDL-C.^[35]^ Abnormal TC levels were significantly positively correlated with the expression regulation network of the renal tubular injury marker KIM-1. Hypercholesterolemia leads to the accumulation of lipid droplets in renal tubular epithelial cells, inhibits cell proliferation, and induces mitochondrial damage, producing reactive oxygen species (ROS) that activate NF-κB and AP-1 and enhance the transcription of KIM-1.^[36,37]^ Hypercholesterolemia also affects the activity of sodium channels (ENaC) by regulating the distribution of phosphatidylinositol-4,5-bisphosphate (PIP2), exacerbating metabolic abnormalities in renal tubular epithelial cells.^[38]^ Moreover, cholesterol overload inhibits lysosomal lipid degradation, leading to autophagy arrest and the death of renal tubular epithelial cells.^[39]^ Under chronic hypercholesterolemia, proximal tubular cells significantly enhance pathological endocytosis through megalin receptors, low-density lipoprotein receptors (LDLR), and scavenger receptors, leading to an imbalance in intracellular free cholesterol homeostasis and causing lipid deposition and lipotoxicity.^[40–42]^ When cholesterol overload exceeds the cellular clearance threshold, endoplasmic reticulum stress may activate the IRE1-XBP1 pathway, bypassing the negative feedback of SREBP-2 and leading to continuous cholesterol synthesis by HMGCR, forming a vicious cycle.^[43]^ Pro-inflammatory factors such as TNF-α downregulate the ATP-binding cassette transporter A1 (ABCA1)/ABCG1-dependent cholesterol efflux mechanism while promoting lysosomal degradation of ABCG1, further weakening cholesterol efflux.^[44]^ Lipotoxicity damages renal tubular epithelial cells through a network of cascades involving induction of inflammation, promotion of fibrosis, and regulation of apoptosis. In terms of inflammation regulation, the activation of the NLRP3 inflammasome and the upregulation of the TLR4/ NF-κB signaling pathway form a synergistic amplification effect.^[45–47]^ In terms of fibrosis, abnormal expression of TGF-β promotes myofibroblast transdifferentiation and excessive deposition of the extracellular matrix (ECM) through Smad-dependent signaling pathways^[48,49]^; in terms of apoptosis regulation, the mitochondrial apoptotic pathway (significant decrease in the Bcl-2/Bax ratio) and the death receptor pathway (activation of the Fas/FasL system) lead to programmed cell death.^[50,51]^ These molecular events ultimately manifest as characteristic pathological changes such as tubular atrophy and interstitial fibrosis at the tissue level.

Hypertriglyceridemia (HTG)-mediated renal tubular injury, particularly in the proximal tubules, exhibits characteristics of multilevel molecular network interactions. The pathological accumulation of free fatty acids, which are hydrolysis products of TG mediated by lipoprotein lipase, in proximal tubule epithelial cells triggers a cascade of pathological reactions: Lipotoxic effects directly drive the transcriptional activation of the KIM-1 gene through the ERK1/2 signaling axis.^[52]^ Saturated fatty acids disrupt endoplasmic reticulum homeostasis to initiate the unfolded protein response core signaling axis (PERK/IRE1α/ATF6), and the terminal effector molecule C/EBP homologous protein (CHOP) may participate in the regulation of KIM-1 expression through epigenetic modifications^[53,54]^; The NF-κB inflammatory signaling cascade is activated by pattern recognition receptors. Pro-inflammatory mediators such as TNF-α directly promote KIM-1 expression through the JAK-STAT signaling module. The mutual reinforcement between the chronic inflammatory microenvironment and profibrotic phenotype of KIM-1 constitutes a vicious pathological cycle^[55,56]^; Metabolic oxidative stress caused by the combined effects of impaired mitochondrial β-oxidation (ATP synthesis) and excessive peroxisomal oxidation (overproduction of reactive oxygen species, ROS) indirectly regulates KIM-1 transcription through the activation of redox-sensitive pathways such as MAPK^[57–59]^; Imbalance in the autophagy-lysosome system flux, acting as a lipotoxicity amplifier, indirectly upregulates KIM-1 expression through the release of apoptosis-related molecular patterns via the mitochondrial pathway (e.g., cytochrome C) and caspase-dependent/independent pathways.^[60–63]^ Systematic deconstruction of this molecular network provides a key theoretical basis for molecular classification and targeted intervention strategies for HTG-related renal tubular lesions.

As the core structural protein of HDL, ApoA1 is defined within the classical theoretical framework as a biomolecule with multiple protective effects including anti-atherosclerosis, regulation of cholesterol reverse transport, and anti-inflammatory and antioxidant properties.^[64,65]^ However, its association with the renal tubular injury marker KIM-1 presents a complex, multidimensional interaction network that may exhibit functional heterogeneity and bidirectional regulatory effects under pathological conditions. The renal protective effects of the ApoA1/HDL system are primarily achieved through the following pathways: clearance of abnormally accumulated lipids in renal tubular cells through cholesterol reverse transport^[66,67]^; inhibition of the activation of inflammatory signaling pathways, such as NF-κB, thereby reducing oxidative stress damage^[68]^; and improvement of endothelium-dependent vasodilation and optimization of renal microcirculatory perfusion.^[69]^ However, under specific pathological conditions, elevated ApoA1 levels may paradoxically correlate with upregulated KIM-1 expression, suggesting a “functional-structural dissociation” phenomenon.^[70–72]^ This phenotypic heterogeneity may be attributed to: accumulation of dysfunctional HDL particles; in pathological states such as CKD and diabetic nephropathy, the proteomic and lipidomic profiles of HDL particles undergo significant changes, resulting in the formation of pro-oxidant or inflammation-sensitive subpopulations with reduced cholesterol efflux capacity and enhanced pro-inflammatory activity^[73,74]^; renal tubular metabolic abnormalities: proximal tubular cells reabsorb ApoA1 through the megalin-cubilin complex-mediated receptor pathway. Disruption of this process may lead to abnormal intracellular accumulation of ApoA1, triggering lysosomal storage injury^[75]^; Post-translational modification effects: Under oxidative stress or high-glucose conditions, ApoA1 is prone to modifications such as tyrosine nitration and methionine oxidation, which significantly reduce its binding capacity to ABCA1 while acquiring pro-inflammatory ligand properties.^[76]^ Notably, based on epidemiological evidence of a U-shaped association between HDL-C and renal prognosis, ApoA1 may exhibit opposite dose-effect characteristics on renal tubular epithelial cells when its concentration is either too high or too low: ApoA1 exerts protective effects within the physiological concentration range (1.20–1.60 g/L),^[77]^ whereas at supraphysiological concentrations (>2.0 g/L), it may exceed the renal tubular metabolic threshold, leading to upregulation of KIM-1 expression through activation of the Toll-like receptor 2/4 (TLR2/4) signaling pathway.^[78,79]^ This biphasic effect suggests that the “quality” (functional activity) of ApoA1 is more pathophysiologically significant than its “quantity” (circulating concentration). Moreover, ApoA1 modified by advanced glycation end products (AGE-ApoA1) may play a unique role in diabetic nephropathy, as it not only loses the ability to promote cholesterol efflux, but also activates the p38 MAPK pathway by binding to the receptor for advanced glycation end products (RAGE), inducing epithelial-mesenchymal transition in renal tubular epithelial cells. This process is closely related to the upregulation of KIM-1 expression and progression of renal interstitial fibrosis.^[52,80]^ In summary, the interaction network between ApoA1 and KIM-1 is significantly dependent on pathological context. When assessing their correlation, it is necessary to systematically consider multiple factors, including the functional status of HDL particles, characteristics of the local renal microenvironment, and genetic and epigenetic modifications. Current evidence suggests that simply increasing circulating ApoA1 concentration does not improve the renal protective effects. Future research should focus on developing HDL function testing systems and targeted therapeutic strategies to repair dysfunctional HDL with the aim of achieving true renal tubular protection.

In this study, no causal relationships were detected between LDL-C, HDL-C, ApoB, and the renal injury marker KIM-1. This finding is particularly noteworthy, as it contrasts with the close associations between lipid metabolism abnormalities and CKD suggested by numerous observational studies.

Numerous epidemiological studies have identified elevated LDL-C, particularly ApoB, which reflects the number of atherogenic lipoprotein particles, as an independent risk factor for the occurrence, progression, and cardiovascular complications of CKD.^[81]^ These studies generally indicate that increased levels of LDL-C and ApoB are significantly associated with an accelerated decline in the estimated glomerular filtration rate (eGFR) and the occurrence and exacerbation of proteinuria, such as elevated albuminuria. Mechanistically, high LDL-C/ApoB not only affects renal perfusion by accelerating systemic atherosclerosis, but more importantly, it can directly damage intrinsic renal cells, including glomerular podocytes, endothelial cells, and PTECs.^[82]^ Considering the direct damaging effects of high LDL-C/ApoB on PTECs, observational studies might theoretically find that elevated LDL-C/ApoB levels are correlated with upregulated expression of KIM-1 in the proximal tubules and increased urinary KIM-1 levels.^[83]^ Multiple cohort studies have shown that ApoB levels are positively correlated with the risk of CKD, especially in patients with hypertension or hyperuricemia, where each 1 g/L increase in ApoB is associated with a 1.3-fold increase in CKD risk,^[81]^ Urinary KIM-1 levels are significantly elevated in patients with increased LDL-C/ApoB levels and are independently correlated with the urinary protein excretion rate (UPCR).^[84]^

Low HDL-C levels are also considered a risk factor for CKD, especially in patients with CKD. In a Korean study involving 2168 patients, those with HDL-C levels below 30 mg/dL had a significantly higher risk of CKD progression than patients with HDL-C levels in the range of 50 to 59 mg/dL (HR = 2.21, 95% CI: 1.33–3.70).^[85]^ There is a close relationship between low HDL-C levels and a decline in eGFR. In one study, low HDL-C levels (<40 mg/dL) were significantly associated with an eGFR below 60 mL/min/1.73 m^2^, and the trend of decreasing HDL-C levels became more pronounced with decreasing eGFR.^[86]^ The protective mechanisms of HDL include reverse cholesterol transport, antioxidant activity, anti-inflammatory effects, and the maintenance of endothelial function.^[87]^ Based on these mechanisms, lower HDL-C levels may render PTECs more susceptible to damage, and may theoretically be associated with upregulated KIM-1 expression. In contrast, high-functional HDL-C may suppress KIM-1 expression by alleviating stress and promoting lipid efflux.^[88]^

The differences between this study and the observational studies can be explained in several ways. First, the specificity of the outcome indicator, KIM-1. KIM-1 is a sensitive biomarker for acute or chronic early injury of renal tubular epithelial cells and mainly reflects cellular stress. In contrast, the pathogenic effects of LDL-C/ApoB are long-term and cumulative, with the main pathological changes likely manifesting as more advanced and extensive damage, such as glomerulosclerosis and renal interstitial fibrosis. These late-stage lesions may initially present as proteinuria or decreased eGFR in the early stages rather than a persistent and significant elevation of KIM-1. Therefore, even if high LDL-C/ApoB levels damage PTECs, such damage may not always be primarily characterized by persistent high-level expression of KIM-1. Similarly, the protective effects of HDL-C may focus more on preventing or delaying overall kidney damage, with relatively smaller or more indirect effects on KIM-1 levels. Second, the role of confounding factors in observational studies was crucial. Populations with high LDL-C/ApoB often have diabetes, hypertension, obesity, and other independent risk factors for CKD, which can damage the renal tubules and potentially affect KIM-1 expression. In observational studies, the association between lipids and KIM-1 is likely to be partly or mainly due to uncontrolled confounding factors. In contrast, MR effectively avoids such biases. Third, the stage of injury is another important consideration. Lipid abnormalities can lead to progressive and long-term kidney damage. Genetic variants represent lifelong exposure, which may lead to pathological changes in later stages of the disease. KIM-1, as a marker of early or active damage, may have higher expression at specific times, and its association with lifelong exposure may not be as direct or strong as its association with long-term outcomes, such as end-stage renal disease. MR may be more suitable for detecting the causal effects of exposure on final long-term outcomes than dynamic markers at a specific stage. Fourth, statistical power is a technical factor that must be considered. Limited outcome GWAS sample sizes may lead to insufficient statistical power to effectively detect the effect, resulting in false negative results. Fifth, different lipid components may play different roles in the different layers and stages of kidney injury. Elevated LDL-C/ApoB levels may primarily cause proteinuria through glomerular damage while also injuring renal tubules, but its causal impact on KIM-1 may not be direct or may occur at different pathological stages or affect other structures. The protective effects of HDL-C may be more extensive; however, its effect on preventing glomerular damage (e.g., reducing proteinuria) may be more pronounced.

This finding does not negate the important role of abnormalities in lipid metabolism in CKD. However, this suggests that the association between lipid metabolism abnormalities and renal tubular injury, as well as its link with KIM-1, may not be as direct or widespread as inferred from observational studies, or may be modulated by other factors. Future research could utilize larger sample-sized KIM-1 GWAS data and meticulously explore the specific causal roles of lipid metabolism abnormalities in different stages of CKD and various renal structures.

## 5. Conclusion

Based on the above research findings, the following important clinical implications can be distilled. First, this study suggests that TG and TC may be potential risk factors for early tubular injury independent of traditional lipid indicators (especially LDL-C). This provides guidance for clinical practice, indicating that in managing high-risk populations for kidney disease, such as those with diabetes and hypertension, attention and monitoring of TG and TC levels should be enhanced in addition to routine monitoring of blood glucose, blood pressure, and LDL-C. Elevated TG and TC levels can help identify subgroups of individuals at risk for tubular injury earlier, which may provide a basis for the development of early intervention strategies. Second, in view of the complexity of the associations with ApoA1 and HDL-C, future genetic studies may attempt to use more sophisticated Mendelian randomization analysis methods (such as multivariable MR) or seek genetic instrumental variants more specifically related to ApoA1/HDL function rather than just plasma levels to more accurately assess their causal effects on KIM-1 and minimize potential biases due to pleiotropy. Ultimately, the most clinically translatable research path lies in conducting rigorously designed randomized controlled clinical trials to evaluate whether clinical intervention strategies targeting the reduction of TG (e.g., using fibrate drugs, ω-3 fatty acids, or intensive lifestyle interventions) or TC can effectively reduce urinary KIM-1 levels in high-risk individuals and ultimately achieve the clinical goal of slowing kidney disease progression.

## Author contributions

**Conceptualization:** Keqin Zhao.

**Data curation:** Keqin Zhao.

**Validation:** Keqin Zhao.

**Visualization:** Keqin Zhao.

**Writing – original draft:** Keqin Zhao.

**Writing – review & editing:** Linlin Qian, Xiaobei Ma.
